# Longitudinal study of foot-and-mouth disease virus in Northern Nigeria: implications for the roles of small ruminants and environmental contamination in endemic settings

**DOI:** 10.1186/s13567-025-01502-2

**Published:** 2025-04-03

**Authors:** Simon Gubbins, Emma Brown, Yiltawe Wungak, Olumuyiwa Oyekan, Adeyinka J. Adedeji, Sandra I. Ijoma, Rebecca B. Atai, Moses O. Oguche, Mark Samson, Banenat B. Dogonyaro, Fabrizio Rosso, Hayley Hicks, Britta A. Wood, Jemma Wadsworth, Nick Knowles, Donald P. King, Anna B. Ludi, Claire Colenutt, Andrew E. Shaw, Georgina Limon, David O. Ehizibolo

**Affiliations:** 1 The Pirbright Institute, Ash Road, Pirbright, Surrey UK; 2 National Veterinary Research Institute, Vom, Plateau State Nigeria; 3 European Commission for the Control of Foot-and-Mouth Disease, Food and Agriculture Organisation of the United Nations, Rome, Italy

**Keywords:** Cattle, sheep, goats, surveillance, foot-and-mouth disease virus, endemic disease environmental sampling

## Abstract

Foot-and-mouth disease (FMD) is a highly contagious disease affecting cloven-hoofed ungulates. This study aimed to enhance our understanding of the role of small ruminants and environmental contamination in the epidemiology and endemicity of FMD. A longitudinal study was conducted between March 2021 and October 2021 in northern Nigeria, where monthly samples were collected from five households, one livestock market and one transhumance location in two local government areas (LGA) identified as being at high risk of FMD. Serum samples (*n* = 783), oral swabs (*n* = 424) and environmental swabs (*n* = 458) were collected and tested for the presence of foot-and-mouth disease virus (FMDV) RNA by rRT-PCR. Serum samples (*n* = 780) were also tested for the presence of antibodies against FMDV non-structural proteins. The proportion of FMDV RNA positive samples increased in all sample types collected in one LGA during the period when an FMD outbreak was reported in the same LGA. In contrast, sero-positive samples did not differ by month but differed between LGAs and amongst species. The force of infection estimated from age-seroprevalence data for each household was significantly lower in goats compared with both cattle or sheep. Five O/EA-3 topotype sequences were obtained from selected FMDV RNA positive samples; findings which support the use of environmental swabs to detect circulating FMDV strains in endemic settings. These results show oral and environmental swabs are suitable sampling methods for early detection at animal and herd level, respectively and provide insights on the role of small ruminants on FMD epidemiology.

**Supplementary Information:**

The online version contains supplementary material available at 10.1186/s13567-025-01502-2.

## Introduction

Foot-and-mouth disease virus (FMDV) is an RNA virus (family *Picornaviridae*, genus *Aphthovirus*) characterised by high genetic and antigenic heterogeneity [1]. FMDV is the causative agent of foot-and-mouth disease (FMD), a highly contagious disease affecting wild and domestic cloven-hoofed ungulates. Globally, small ruminants (sheep and goats) represent the largest population of FMD susceptible domestic livestock. Although small ruminants can act as silent shedders of FMDV, their role in the epidemiology of FMD is generally neglected and poorly understood, partly due to the inapparent nature of clinical disease in these hosts [2, 3]. Vesicular signs in small ruminants are often subtle and less apparent than those observed in cattle or are absent [3]. Furthermore, common clinical signs of FMD in small ruminants (lameness, fever, depression) are not disease specific.

In Nigeria, small ruminants account for 84.5% of total grazing domestic livestock (48.6 million sheep and 76.3 million goats) [4]. Sheep and goats are mainly of indigenous breeds and largely kept in mixed farming with cattle by pastoralist and subsistence farmers, predominantly in northern parts of the country. FMD is endemic in Nigeria and, as in other endemic countries, outbreaks are reported mainly in cattle. However, FMDV RNA has been detected in sera and epithelial tissues, and evidence of antibodies against FMDV non-structural proteins (NSP) antibodies has been reported in sheep and goats [5–9]. Higher seroprevalence has been reported in sheep compared to goats [5, 9], and lower seroprevalence in sheep compared to cattle [9]. Serotypes O, A, SAT 1 and SAT 2 have been isolated and characterised from cattle samples in Nigeria [7, 10–12]. These four serotypes belong to diverse topotypes or genotypes and are phylogenetically related to strains circulating in West, Central and Northern Africa [8, 11, 13, 14].

Virus isolation from clinically affected animals is considered the gold standard to confirm that animals are infected with FMDV. This is often followed by antigen ELISA and sequencing of viral genomes to determine the FMDV serotype and topotype circulating in the area. However, these methods have specific laboratory requirements, and depend on farmers or field vets to identify and correctly sample clinically affected animals. This is particularly challenging in endemic countries with scarce resources and where multiple FMDV serotypes circulate. Therefore, alternative, yet reliable, methods are needed to efficiently identify infected animals in a timely manner, conduct outbreak investigations and characterise the circulating serotype. Oral and nasal swabs have been suggested as an alternative method to recover viral RNA from infected and clinically healthy animals [15, 16]. These have the advantages of being less invasive compared to probang sampling and allow the detection of infected animals prior to the appearance of clinical signs and/or when clinical signs are not noticeable.

Environmental sampling (i.e., taking swabs of any surfaces likely to have been contaminated by secretions and excretions of infected animals) also presents an opportunity for non-invasive sample collection, enabling FMD surveillance at herd level and beyond regular investigation of clinical cases [17–19]. This is of particular value in places where sampling individual animals is challenging or not feasible. In addition, environmental sampling presents an alternative to the collection of clinical samples, potentially allowing FMDV detection at herd level faster [20], is less stressful for the animals, and potentially more cost effective. Combining recovery of viral RNA from contaminated surfaces with sequencing of viral genomes allows for strain identification and outbreak tracing.

The aims of this study were (i) to enhance our understanding of the role of small ruminants and environmental contamination in the maintenance of FMD, and (ii) to identify reliable and convenient sampling methods for surveillance in endemic settings.

## Materials and methods

### Study period and location

**Figure 1:**
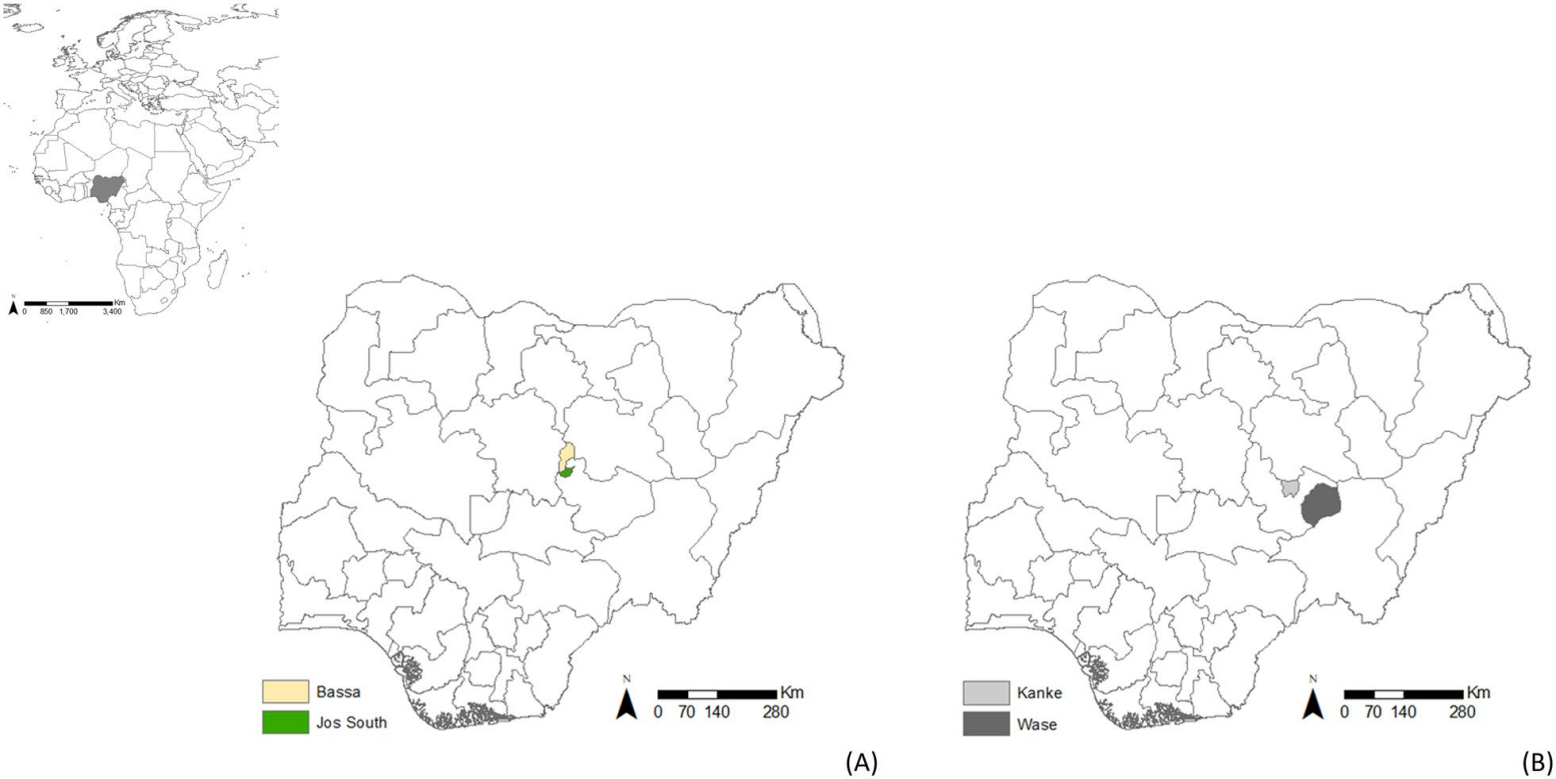
**Maps showing the location of the study areas.** **A** Bassa and Jos South local government areas (study sites) in Plateau State, Nigeria where sampling for foot-and-mouth disease virus was carried out between March and October 2021; and **B** Kanke and Wase local government areas in Plateau State where foot-and-mouth disease outbreaks were reported during the study period.

Samples were collected once a month from March 2021 to October 2021, apart from August 2021 when sampling was not possible for security reasons. Samples were collected from five households, one livestock market and one transhumance location in both Bassa and Jos South local government areas (LGAs) in Plateau State in northern Nigeria (Figure 1A). These LGAs had been identified as being at high risk of FMD based on serological testing of samples from small ruminants [5] and FMD outbreaks reported in 2020 [21]. To be eligible for recruitment, households had to raise both cattle and small ruminants (sheep and/or goats) and agree to participate in the study. Eligible households were identified and selected with input from local contacts in each LGA. All were subsistence farmers keeping mixed herds with indigenous or mixed breeds, often taking animals to communal grazing and water points during the day. Participation was voluntary and no incentives were given to take part in the study. Transhumance locations were defined as a location where herders settle for up to two weeks before continuing their journey. There is only one livestock market and transhumance site in Jos South. Livestock markets and transhumance sites in Bassa were selected based on location, access, and agreement from people in charge to take part in the study. For transhumance sites, animal availability at the time of the sampling was also a factor.

Further samples were collected, by the same field teams, from households reporting clinical outbreaks in the study area and in neighbouring LGAs during the same study period.

In both Bassa and Jos South, the households that were sampled changed between the March and April visits, after which samples were taken from the same households on all subsequent visits (i.e. May–October). Results are presented for all households, but statistical analysis was only carried out using the results from samples collected between April and October.

### Animal sampling

During the first household visit nine animals (3 sheep, 3 goats and 3 cattle) were selected systematically for sampling. If fewer than three animals of a species were present, the total number was completed by sampling other species. At subsequent visits, the same animals were sampled, if possible, but this was often not the case as some animals were sold or slaughtered during the study period. At livestock markets and transhumance sites, animals were selected at each visit from various locations within the market or site. Each animal sampled was given a unique ID number which was linked to the site where the animal was held during the month of the visit. In addition, the age and sex of animals sampled were collected. Clinical examination was conducted on the animals sampled by a qualified veterinarian in the field team, and animals showing FMD-like lesions on the day of the visit were recorded.

Five millilitres of blood were collected from the jugular vein of each selected animal using pre-labelled vacutainer tubes (Becton Dickinson, USA). A sterile swab stick (SkyHealth, China) was used to swab the oropharyngeal/oral cavity of an animal and immediately put into PBS, as the transport medium. Once an animal had been sampled it was identified with a line in the ear using a non-toxic colour marker to avoid double sampling. All samples were kept at 4 °C and sent to the National Veterinary Research Institute (NVRI) in Vom as soon as possible. On arrival at NVRI serum was separated into Eppendorf tubes clearly labelled with the unique animal ID number. Individual serum samples and oral swabs were stored at -20 °C and shipped on dry ice to The Pirbright Institute, UK for testing.

### Environmental sampling

At each sampling site, electrostatic dust cloths were used to swab areas of the environment where contact with secretions and excretions from infected animals was deemed likely (e.g. food troughs, hard floor surfaces, boots and tether ropes, transport vehicles and herder’s sticks). Up to ten environmental samples per site per visit were collected. Each environmental sample was given a unique ID number which was linked to the site, place from which the sample was collected and month of the visit. The environmental samples were processed in the field by eluting the swabs in PBS and then adding the samples directly into lysis buffer (MagMAX Core or RLT buffer, Thermo Fisher Scientific, UK) at a ratio of 1:1. All samples were stored at 4 °C and shipped on dry ice to The Pirbright Institute for testing.

### Sample processing

Environmental samples, serum samples and oral swabs were tested for the presence of FMDV RNA by rRT-PCR. Viral RNA was extracted from samples using the KingFisher Flex automated extraction platform (Thermo Fisher Scientific, UK) with the MagMAX™ CORE Nucleic Acid Purification Kit (Thermo Fisher Scientific, UK). FMDV RNA was detected by rRT-PCR on the ABI 7500 system (Applied Biosystems, UK) using an assay that targets the 3D region of the FMDV genome (forward: ACTGGGTTTTACAAACCTGTGA, reverse: GCGAGTCCTGCCACGGA, probe: TCCTTTGCACGCCGTGGGAC) [22]. Serum samples were also tested for the presence of antibodies against FMDV non-structural proteins (NSP). Samples were heat inactivated (at 56 °C for 30 min, using a heat block) before testing with a PrioCHECK FMDV NS Antibody ELISA kit (Thermo Fisher Scientific Prionics AG, Waltham, MA, USA). Kits were used as per the manufacturer’s instructions.

### Sequencing

Selected FMDV-positive samples were subjected to probe-enriched Illumina-based next generation sequencing. The selected samples had a mean rRT-PCR C_T_ value of 27.2 (range: 20.9 to 32.9; see Additional file 1 for sample details). First and second strand synthesis of total nucleic acid was performed as described previously using the SuperScript™ double-stranded cDNA synthesis kit (ThermoFisher) [23]. Libraries were prepared following the Illumina DNA prep with enrichment protocol. The first stage libraries were pooled and subsequently enriched using a library comprising 26,275 unique biotinylated oligos designed using 622 complete genomes available in GenBank [24]. Final libraries were diluted and run on the Illumina MiSeq using a V2 300 nano cartridge generating 2 × 150 paired-end reads.

Reads were initially assembled into contigs using SPADES, and each contig in turn was queried against a database of FMDV sequences to identify FMDV-specific contigs. Each FMDV contig was further subjected to BLAST online to identify the closest related sequence available. The 1D region of each most closely related virus was selected and used as a reference sequence for the reference assembly using BWA-MEM of each sample. Consensus sequences were extracted following reference assembly using VCF tools. The evolutionary history was inferred by using the Maximum Likelihood method based on the Tamura 3-parameter model [25]. The tree with the highest log likelihood is shown. The percentage of trees in which the associated taxa clustered together is shown next to the branches. Initial tree(s) for the heuristic search were obtained automatically by applying Neighbor-Join and BioNJ algorithms to a matrix of pairwise distances estimated using the Maximum Composite Likelihood (MCL) approach, and then selecting the topology with the superior log likelihood value. A discrete Gamma distribution was used to model evolutionary rate differences among sites [5 categories (+ G, parameter = 0.5492)]. The tree was drawn to scale, with branch lengths measured in the number of substitutions per site. The analysis involved 20 nucleotide sequences. All positions with less than 95% site coverage were eliminated (i.e. fewer than 5% alignment gaps, missing data, and ambiguous bases were allowed at any position). Evolutionary analyses were conducted in MEGA7 [26].

### Statistical analysis

#### Test performance

McNemar's test for paired data and the kappa statistic were used to assess whether the results for oral swabs and serum samples tested by rRT-PCR from the same animals differed significantly from one another. A Bayesian latent class analysis was used to estimate the diagnostic sensitivity (Se) and specificity (Sp), under field conditions, for oral swabs and serum samples tested by rRT-PCR. For this, we adapted a previously published Hui-Walter model [27] implemented using JAGS and *rjags* R package [28, 29] and R (version 4.4.0) [30]. Priors for Se and Sp were informed by previously published values (see Additional files 2 [22, 31, 32] and 3 for details). A beta distribution with parameters α and β was assumed for the priors, and distribution parameters were estimated using the functions *fitdist* and *epi.betabuster* from the R packages *fitdistrplus* and *epiR* [33, 34]. Test results were classified as either positive or negative, and animals were classified as originating from two subpopulations: households or livestock markets and transhumance sites.

#### Proportion of positive samples

The proportion of positive samples by rRT-PCR or by ELISA was analysed using a binomial generalised linear mixed model with a logit link function. Because of the marked difference in the number of positive rRT-PCR results between LGAs, data from Bassa and Jos South were analysed in separate models (see Additional file 4 for details of the models). By contrast, the number of positive ELISA results were similar in both LGAs and, accordingly, the ELISA results were analysed in a single model (see Additional file 4 for details of the models). In each model the response variable was whether or not a sample was positive. Explanatory variables considered in the models included the LGA, month the sample was taken, sample type and species as fixed effects, and sampling location as a random effect. Models were implemented in a Bayesian framework using OpenBUGS (version 3.2.3). Diffuse priors were used for model parameters: normal with mean 0 and standard deviation 10 for regression coefficients; and exponential with mean 100 for the random effect variance. Two chains of 60 000 iterations were run with the first 10 000 samples discarded to allow for burn-in of the chains. Chains were subsequently thinned by selecting every fifth iteration to reduce autocorrelation amongst the samples. Convergence was monitored visually and using the Gelman-Rubin statistic in OpenBUGS. Models including different explanatory variables were compared using the deviance information criterion (DIC) [35].

#### Basic reproduction number for FMDV

The basic reproduction number (*R*_0_) was estimated in two ways, reflecting what is measured by the diagnostic tests used to infer *R*_0_. First, we used the NSP ELISA data to estimate the force of infection (i.e. the rate at which susceptible individuals acquire infection) for each household and, hence, *R*_0_, from age-seroprevalence data. This is not a measure of the first exposure of an animal to FMDV, because NSP ELISA reflects historic exposure to any strain or serotype of FMDV rather than recent seroconversion. Second, we used the rRT-PCR data for serum samples and oral swabs to estimate *R*_0_ for individual outbreaks in households. This reflects the fact that rRT-PCR measures only recent exposure to FMDV, though the PCR target (3D gene of FMDV) used in the present study is not strain or serotype specific.

To estimate the force of infection for each household from NSP ELISA data, the age and NSP status of each animal (i.e. negative or positive by NSP ELISA) at its first sampling was extracted from the data (see Additional files 5 and 6 for results for individual animals). To simplify the analysis, only home-bred animals (*n* = 306) were included in the analysis; those bought-in to the household (*n* = 18) were excluded. The force of infection was estimated using a catalytic model [36, 37], so that

$$p\left(a\right)=1-\text{exp}\left(-\lambda a\right),$$

where *p*(*a*) is the seroprevalence at age *a* and *λ* is the force of infection. The force of infection can then be used to estimate the basic reproduction number, *R*_0_, using the relationship *R*_0_ = *λL* where *L* is the mean lifespan of an animal [37]. For cattle and sheep in the study area, the mean lifespans are 5 and 2 years, respectively.

Heterogeneities in the force of infection were incorporated to allow it to vary amongst species and households/LGAs. In this case, the force of infection for an individual is given by

$$\text{log}\lambda =\alpha +{\beta }_{SPP}+{\gamma }_{LGA,HOUSE},$$

where *α* is the baseline, *β* is the effect of species and *γ* is the effect of LGA and household. Here γ is treated as a random effect and drawn from a normal distribution with mean 0 and standard deviation *σ*_*γ*_.

Parameters in the model were estimated in a Bayesian framework using OpenBUGS (version 2.3.2). A Bernoulli likelihood was used with diffuse priors for model parameters (normal with mean 0 and standard deviation 10 for *α* and *β*; exponential with mean 100 for *σ*_*γ*_). Two chains of 30 000 iterations were run with the first 5000 samples discarded to allow for burn-in of the chains. Chains were subsequently thinned by selecting every fifth iteration to reduce autocorrelation amongst the samples. Convergence was monitored visually and using the Gelman-Rubin statistic in OpenBUGS. Models including different explanatory variables were compared using the DIC (see Additional file 7 for details of the models). The final model was checked by comparing the observed data to the posterior predictive distribution [38].

To estimate the basic reproduction number for individual outbreaks, we used the results of testing serum samples and oral swabs by rRT PCR collected from cattle and sheep. An outbreak was defined as any one or two consecutive monthly samplings when at least one sample from the household was positive by rRT-PCR (note: there were no three or more consecutive monthly samplings with positive PCR results). The proportion of animals positive by rRT-PCR was assumed to be an estimate of the final size of the outbreak. The final size of an outbreak, *f*, is related to the basic reproduction number, *R*_0_, by the following equation,

$$1-f-{s}_{0}\text{exp}\left(-{R}_{0}f\right)=0,$$

where *s*_0_ is the initial proportion of the household that is susceptible [39].

Parameters (i.e. *R*_0_ and *s*_0_ for each outbreak) were estimated in a Bayesian framework. A binomial likelihood was used for the data, that is,

$$L=\prod_{h}{f}_{h}^{{P}_{h}}{(1-{f}_{h})}^{{N}_{h}-{P}_{h}},$$

where *f*_*h*_ is the final size and *P*_*h*_ and *N*_*h*_ are the number of PCR positive animals (based on serum samples and oral swabs) and the number of animals tested, respectively, for outbreak *h*. A gamma prior with mean 10 and shape 2 was used for *R*_0_, while a uniform prior with range [0,1] was used for *s*_0_. Samples from the joint posterior distribution were generated using an adaptive Metropolis algorithm [40], modified so that the scaling factor was tuned during burn-in to ensure an acceptance rate of between 20 and 40% for more efficient sampling of the target distribution [41]. Two chains of 600 000 iterations were run with the first 100 000 samples discarded to allow for burn-in of the chains. Chains were subsequently thinned by selecting every fiftieth iteration to reduce autocorrelation amongst the samples. The adaptive Metropolis scheme was implemented using Matlab (version 2020b; The Mathworks, Inc.). Convergence was monitored visually and using the Gelman-Rubin statistic in the coda package [42] in R (version 4.4.0) [30]. The final model was checked by comparing the observed data to the posterior predictive distribution [38].

To explore differences in *R*_0_ between cattle and sheep for individual outbreaks, we carried out three analyses: one using data for cattle and sheep combined; one using data for cattle only; and one using data for sheep only.

## Results

### Animal sampling for FMDV RNA

**Figure 2:**
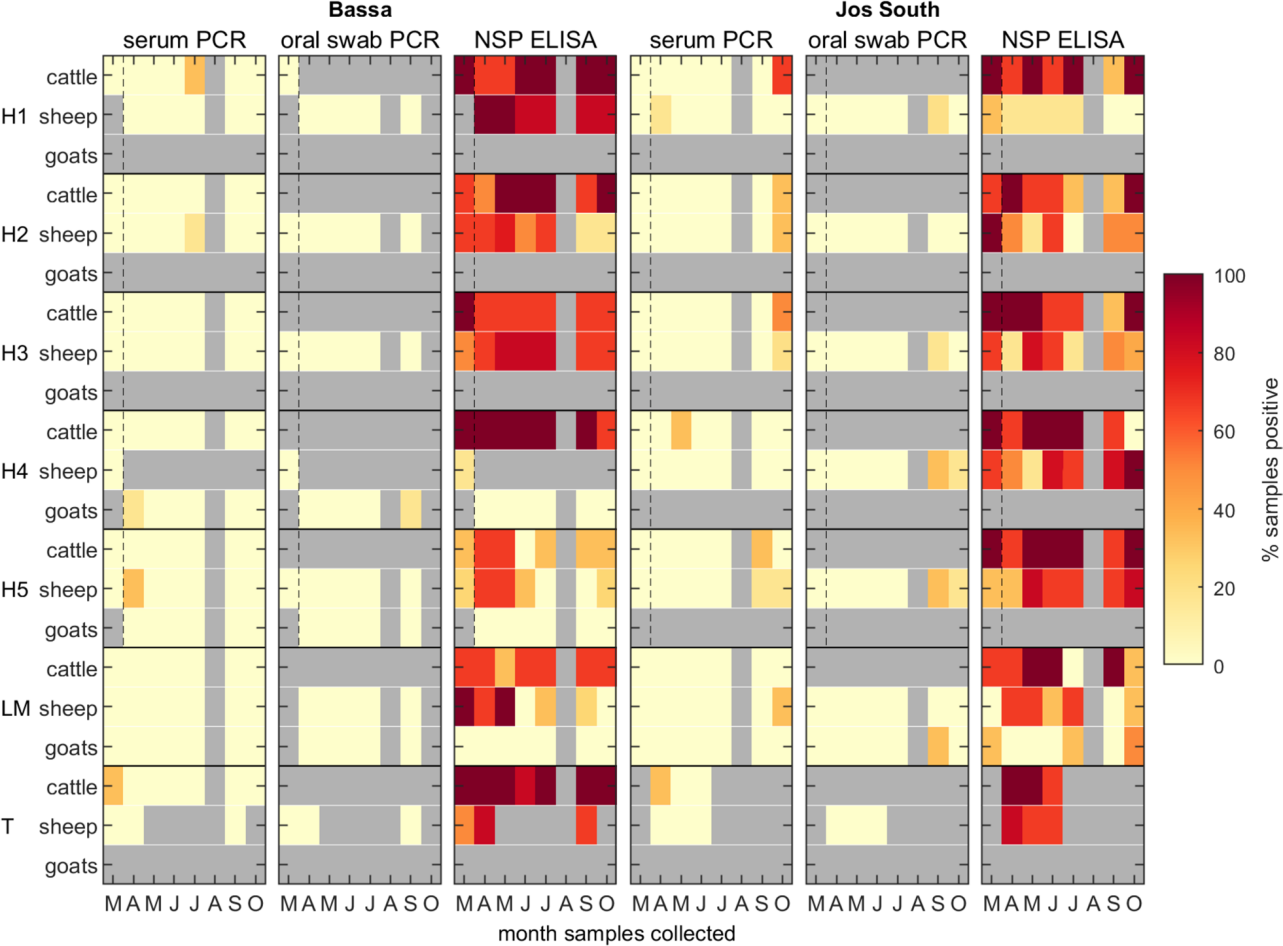
**Longitudinal oral swab and serum sampling for the presence of foot-and-mouth disease virus (FMDV) in cattle, sheep and goats in northern Nigeria between March and October 2021.** FMD viral genome was detected by rRT-PCR (PCR), and antibodies to FMDV non-structural proteins (NSP) were detected by ELISA. Sera and oral swabs were paired (i.e. collected from the same animal). Samples were collected from animals at households (H1-H5), one livestock market (LM) and one transhumance location (T) in both Bassa and Jos South local government areas. Coloured shading indicates the proportion (%) of positive samples; grey shading indicates that no samples were collected. Note: households sampled in March are not the same as those sampled in April–October.

A total of 783 serum samples were collected (591 from animals in households, 126 in livestock markets and 66 from transhumance sites) and tested for FMDV RNA by rRT-PCR (Table 1). Serum samples were collected from cattle (*n* = 284; 215 females and 69 males), sheep (*n* = 407; 332 females and 75 males) and goats (*n* = 92; 68 females and 24 males), of which nine (3.2%), nine (2.2%) and one (1.1%) were positive, respectively (Figure 2; see also Additional files 5 and 6 for results for individual animals). The median age of animals sampled was 5 years (interquartile range (IQR): 3–5 years) in cattle; 2 years (IQR: 1–3 years) in sheep; and 2 years (IQR: 1–2.5 years) in goats. Between zero and four (0%-3.2%) serum samples were positive each month, except for October where nine (8.3%) were positive (Table 1). Positive samples were collected at households and transhumance locations in both Bassa and Jos South and at the livestock market in Jos South (Figure 2).

**Table 1:** **Summary of environmental, serum and oral swab samples tested by rRT-PCR for foot-and-mouth disease virus (FMDV) RNA or for antibodies against FMDV non-structural proteins (NSP) by ELISA**

Month	Environmental swabs		rRT-PCR				NSP ELISA	
			Oral swabs		Serum samples		Serum samples	
	Tested	Positive	Tested	Positive	Tested	Positive	Tested	Positive
March	71	0	38	0	90	1 (1.1%)	90	55 (61.1%)
April	75	2 (2.7%)	77	0	125	4 (3.2%)	125	72 (57.6%)
May	77	0	71	0	117	1 (0.9%)	117	73 (62.4%)
June	75	0	71	0	123	0	122	71 (58.2%)
July	74	0	68	0	111	2 (1.8%)	110	56 (50.9%)
August^a^	0	–	0	–	0	–	0	–
September	56	15 (26.8%)	65	7 (10.8%)	108	2 (1.9%)	108	51 (47.2%)
October	30	1 (3.3%)	34	2 (5.9%)	109	9 (8.3%)	108	57 (52.8%)
total	458	18 (3.9%)	424	9 (2.1%)	783	19 (2.4%)	780	435 (55.8%)

^a^No sampling was carried out in August for security reasons.

Oral swabs were collected from a subset of the animals from which serum samples were taken (*n* = 424) and tested for FMDV RNA by rRT-PCR (Table 1). Samples were collected from cattle (*n* = 3; all female), sheep (n = 345; 278 females and 67 males) and goats (*n* = 76; 55 females and 21 males), of which zero (0%), seven (2.0%) and two (2.6%) were positive, respectively (Figure 2; see also Additional files 5 and 6 for results for individual animals). All cattle sampled were 4 years old. The median age for sheep sampled was 2 years (IQR: 1–3 years), and for goats sampled was 2 years (IQR: 1–2.5 years). None of the oral swabs collected between March and July were positive for FMDV RNA, but nine collected during September and October were positive (Table 1). Of these positive samples, one was collected at a household in Bassa (a female goat, 18 months old), while the remaining eight were collected at four (of five) households (all sheep, seven females and one male) and at the livestock market (one female goat, 18 months old) in Jos South (Figure 2).

Although there was a no significant difference between the proportion of positives (*p* > 0.9), there was only a slight agreement (kappa = 0.08) between serum and oral swabs tested for FMDV RNA (see Additional file 8 for further details). FMDV RNA is detected only for a short period of time (both in serum samples and oral swabs), with a slight difference in timing and short period of overlap. Infected animals are likely to test positive by rRT-PCR in oral samples first, and slightly later in serum (Figure 4) once the infection is systemic. Sensitivity and specificity were estimated (posterior median and 95% credible interval (CI)) to be 0.720 (0.551–0.855) and 0.980 (0.964–0.990) for rRT-PCR in serum, and 0.877 (0.780–0.943) and 0.979 (0.963–0.993) for rRT-PCR in oral swabs.

### Clinical signs of FMD

One two-year old bovine from household H5 in Jos South showed clinical signs of FMD during the sampling visit in September. A serum sample taken from the animal was positive for viral RNA (C_T_ value: 26.6) and negative by NSP ELISA. Two further cattle, one sampled in March (from household H2 in Bassa) and one sampled in October (at the livestock market in Bassa) also showed FMD-like signs, but serum samples from both animals were negative for viral RNA and by NSP ELISA.

### Environmental sampling for FMDV RNA

**Figure 3:**
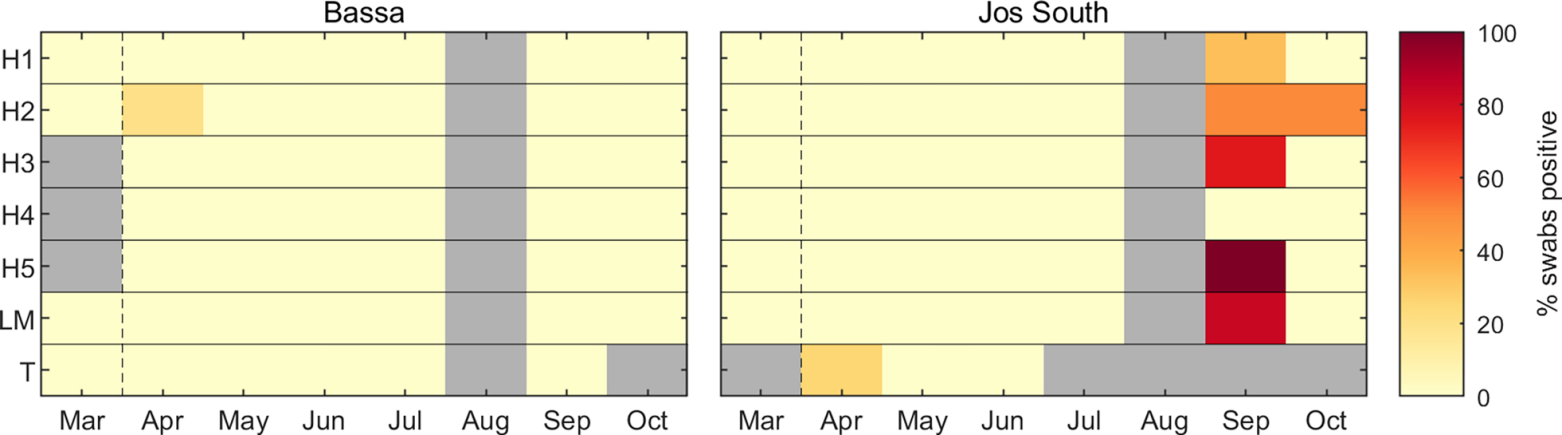
**Longitudinal environmental sampling for the presence of foot-and-mouth disease virus (FMDV) in northern Nigeria between March and October 2021.** Samples collected from households (H1-H5), one livestock market (LM) and one transhumance location (T) in both Bassa and Jos South local government areas. Coloured shading indicates the proportion (%) of samples positive for FMDV RNA by rRT-PCR; grey shading indicates that no samples were collected. Note: households sampled in March are not the same as those sampled in April–October.

A total of 458 environmental samples were collected (Table 1). For the samples collected between March and July, only two (0.5%) were positive for FMDV RNA (Table 1; Figure 3). One positive swab was from a herder’s stick at a household in Bassa, and the other was from boots at a transhumance site in Jos South. By contrast, 16 (out of 86; 18.6%) environmental samples collected in September and October were positive for FMDV RNA (Table 1). These were all collected in Jos South, at four (of five) households and the livestock market (Figure 3). Positive samples were collected from boots (*n* = 4), ropes (*n* = 3), pegs (*n* = 3), transport vehicles (*n* = 2) and hard floor surfaces (*n* = 4).

### Comparison of animal and environmental sampling for FMDV RNA

**Figure 4:**
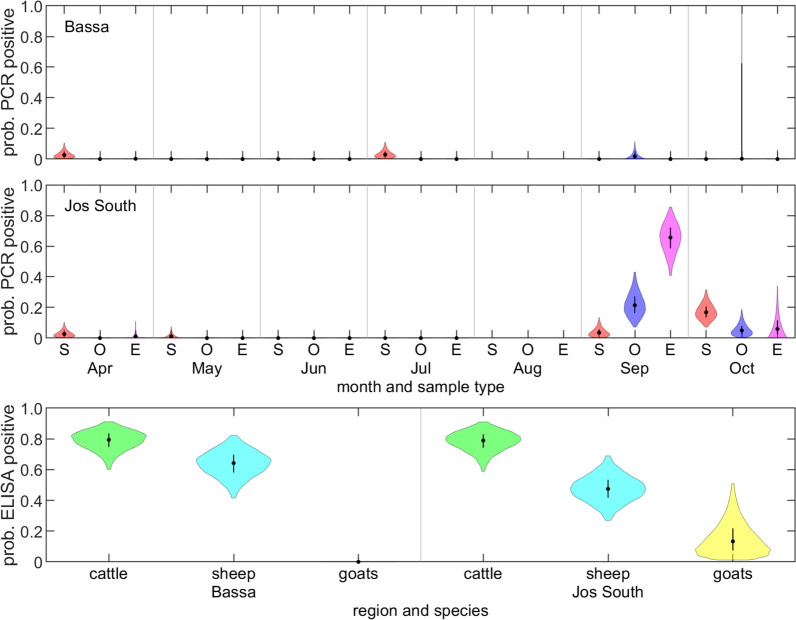
**Probability of a sample being positive for foot-and-mouth disease virus (FMDV) RNA by rRT-PCR or antibodies against FMDV non-structural proteins (NSP).** Samples were collected during a longitudinal study in Bassa and Jos South local government areas between April and October 2021. Sample types tested by rRT-PCR were serum (S, red), oral swabs (O, blue) and environmental swabs (E, magenta). Samples tested by NSP ELISA were from cattle (green), sheep (cyan) or goats (yellow). Violin plots show the posterior median (circle), interquartile range (line) and density (shape) for the probability.

Based on changes in DIC the proportion of positive samples in both regions differed by month and amongst sample types (see Additional file 4 for details of the models and their DICs). Specifically, the proportion of positive samples differed amongst environmental swabs, oral swabs and serum samples, but not with the species the animal samples were collected from. For samples collected in Bassa, the proportion of positive samples was low (posterior median < 2.9%) for all months and sample types (Figures 2–4). For samples collected in Jos South, the proportion of positive samples was low (posterior median < 2.6%) for all sample types collected between May and July, and then increased (posterior median > 3.5%) for samples collected in September or October (Figure 4). In September, the proportion of positive samples was highest for environmental swabs, followed by oral swabs, then serum samples (posterior medians of 65.6%, 21.4% and 3.5%, respectively). In October, this pattern changed, with the highest proportion of positive samples in serum samples, followed by environmental swabs and then oral swabs (posterior medians of 16.8%, 5.9% and 5.0%, respectively). This pattern could be related to FMD outbreaks reported in LGAs near to the study area (see below).

### Antibodies to FMDV non-structural proteins (NSP)

A total of 780 serum samples (283 from cattle, 406 from sheep and 91 from goats) were tested by ELISA for NSP antibodies, of which of which 219 (77.3%), 212 (52.2%) and three (3.3%) were seropositive for cattle, sheep and goats, respectively. Seropositive animals were detected throughout the study period with between 47.2% and 62.4% of animals seropositive each month (Table 1; Figure 2; see also Additional files 5 and 6 for results for individual animals). For animals that were repeatedly sampled at different households, animals that seroconverted for the first time during the study period tend to remain seropositive for the rest of the study period, which is expected for FMDV-NSP antibodies. For those animals that became seronegative during the study period, it was unknown how long they had been seropositive, as they were already seropositive at the time of the first sampling.

Based on changes in DIC the proportion of NSP ELISA positive samples did not differ by month but did differ between regions and amongst species (see Additional file 4 for details of the models compared and their DICs). In both regions, the proportion of NSP ELISA positive samples was highest in cattle, followed by sheep, and lowest in goats (Figure 4). The proportion of seropositive cattle did not differ between regions (posterior median: 79.4% and 78.9% for Bassa and Jos South, respectively). However, the proportion of seropositive sheep was lower in Jos South compared with Bassa (posterior median: 64.3% and 47.5% for Bassa and Jos South, respectively), while the proportion in goats was lower in Bassa compared with Jos South (posterior median: < 0.1% and 13.3% for Bassa and Jos South, respectively).

### Basic reproduction number for FMDV

**Figure 5:**
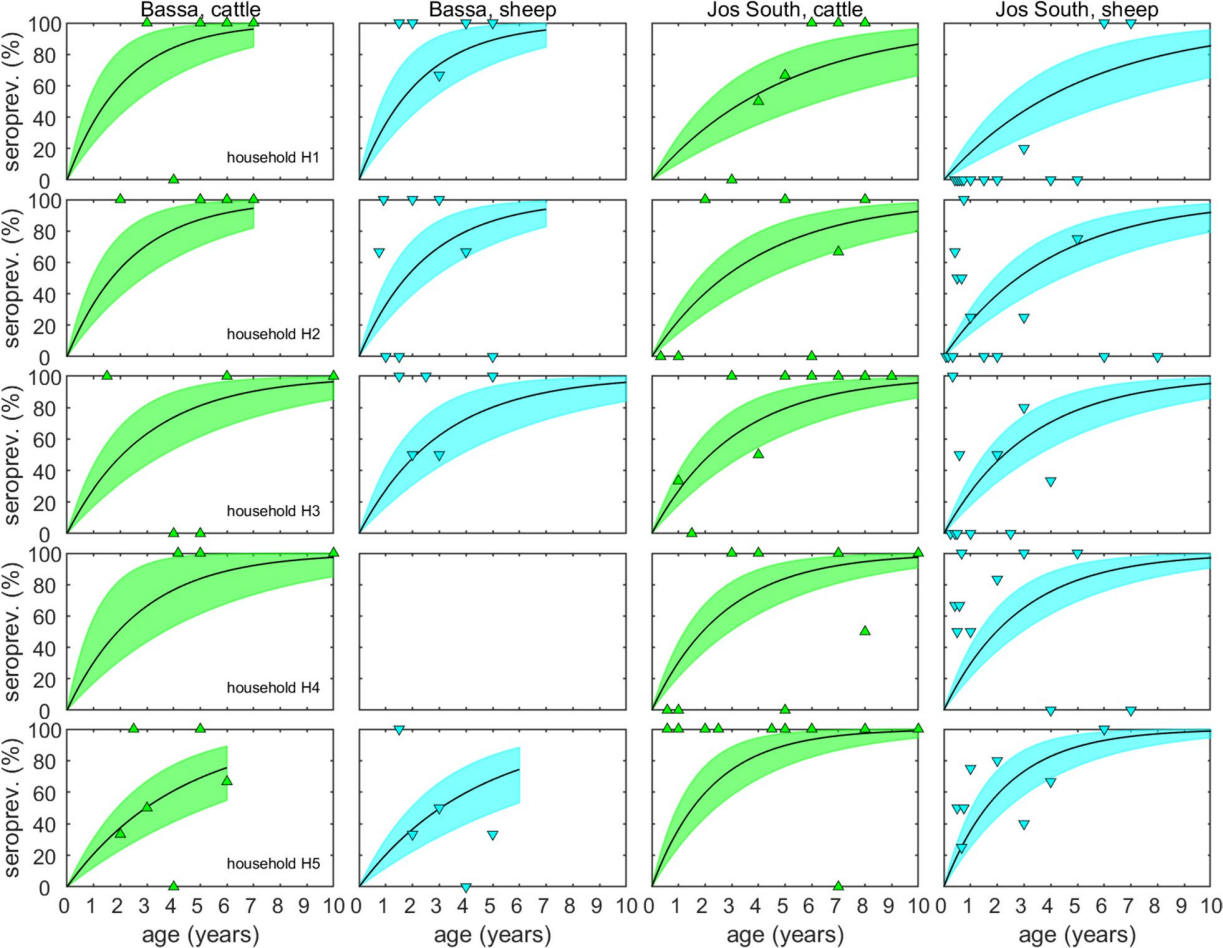
**Relationship between age and seroprevalence for foot-and-mouth disease virus (FMDV) in cattle and sheep, by household, in Bassa and Jos South local government areas.** Each plot shows the observed seroprevalence (i.e. proportion (%) of animals positive for antibodies to FMDV non-structural proteins by ELISA) at each age (triangles) and the expected seroprevalence based on the estimated force of infection (posterior median: black line; 95% credible region: coloured shading) for cattle (green) and sheep (cyan).

Seroprevalence increased with age for both cattle and sheep in all five households sampled in Bassa and Jos South (Figure 5). Goats were only sampled in two households (H4 and H5) in Bassa, all of which were negative by NSP ELISA (Figure 2). Changes in DIC show that the corresponding force of infection differed amongst species and amongst households (see Additional file 7 for details of the models compared and their DICs). Posterior predictive checking showed that the model for the seroprevalence data provided an acceptable fit to the data, with all but one observation lying in the 95% prediction intervals (see Additional file 9 for model checking).

The force of infection in goats was significantly lower than that in cattle or sheep (goats relative to cattle (posterior median and 95% CI: 1.8 × 10^–4^ (9.1 × 10^–11^-0.082); goats relative to sheep: 1.9 × 10^–4^ (1.0 × 10^–10^-0.085); Table 2). However, the force of infection in sheep was comparable to that in cattle (that in sheep relative to cattle: 0.96 (0.68–1.39); Table 2). The force of infection differed amongst households, but there were no systematic differences between the two regions (Figure 5). The basic reproduction number (*R*_0_) for cattle and sheep was calculated from the force of infection by multiplying it by the mean lifespan of the species. This indicated that the basic reproduction number was higher for cattle (median: 1.75; range: 1.00–2.31) than for sheep (median: 0.68; range: 0.39–0.89) across all ten households (Table 3). For all ten households the median *R*_0_ for cattle was ≥ 1 and for four households it was significantly (*P* < 0.05) ≥ 1 (Table 3). By contrast, the median *R*_0_ for sheep was < 1 for all ten households and for four households it was significantly (*P* < 0.05) < 1 (Table 3).

**Table 2:** **Estimated parameters for the force of infection for foot-and-mouth disease virus in households in Northern Nigeria**

Parameter^a^	Posterior median	95% credible limit	
		Lower	Upper
Baseline (*α*)	− 1.11	− 1.47	− 0.73
Species (*β*)			
Cattle	0 (baseline)	–	–
Sheep	− 0.04	− 0.38	0.33
Goats	− 8.60	− 23.12	− 2.50
Household random effect (*σ*_*γ*_)	0.34	0.11	0.70

^a^Note that these parameters and on the natural logarithm scale.

**Table 3:** **Basic reproduction number (*R***_**0**_**) for foot-and-mouth disease virus in households in Northern Nigeria**

Region	Household	Outbreak	Cattle			Sheep			Cattle and sheep		
			Estimate*	95% credible limit		Estimate	95% credible limit		Estimate	95% Credible limit	
				Lower	Upper		lower	upper		Lower	Upper
PCR results for individual outbreaks											
Bassa	H1	July	1.07	0.21	2.57	–	–	–	0.63	0.12	1.20
	H2	July	–	–	–	0.71	0.13	1.43	0.64	0.12	1.18
	H5	April	–	–	–	1.06	0.20	2.51	0.73	0.14	1.42
Jos South	H1	April	–	–	–	0.72	0.14	1.43	0.63	0.11	1.19
		Sep-Oct	1.01	0.20	2.16	0.61	0.12	1.14	0.67	0.13	1.19
	H2	Sep-Oct	0.78	0.15	1.63	0.75	0.15	1.43	0.71	0.14	1.26
	H3	Sep-Oct	0.71	0.13	1.43	0.61	0.11	1.16	0.61	0.12	1.11
	H4	May	1.07	0.20	2.55	–	–	–	0.62	0.12	1.19
		Sep-Oct	–	–	–	0.69	0.13	1.28	0.61	0.11	1.10
	H5	Sep-Oct	0.71	0.13	1.44	0.84	0.16	1.53	0.72	0.15	1.29
Kanke	P7	Dec	–	–	–	2.05	0.51	4.40	–	–	–
Age-dependent seroprevalence (NSP ELISA)											
Bassa	H1	–	2.31	1.34	4.36	0.89	0.53	1.64	–	–	–
	H2	–	1.00	0.55	1.64	0.39	0.21	0.62	–	–	–
	H3	–	2.06	1.22	3.70	0.80	0.50	1.34	–	–	–
	H4	–	1.30	0.80	1.96	0.50	0.32	0.73	–	–	–
	H5	–	1.67	0.96	2.85	0.65	0.37	1.09	–	–	–
Jos South	H1	–	1.58	0.99	2.47	0.61	0.39	0.92	–	–	–
	H2	–	1.82	0.95	4.45	0.70	0.37	1.70	–	–	–
	H3	–	1.82	1.19	2.83	0.70	0.47	1.05	–	–	–
	H4	–	1.17	0.66	1.85	0.46	0.25	0.72	–	–	–
	H5	–	2.27	1.44	3.63	0.88	0.58	1.34	–	–	–

Ten outbreaks were identified during the study period, where an outbreak was defined as any one or two consecutive monthly samplings when at least one sample was positive by rRT-PCR. Three outbreaks occurred in households in Bassa and five in households in Jos South (Figure 2). Six of the households had a single outbreak, while two (both in Jos South) had two outbreaks each with gaps of three or four months between outbreaks (Figure 2). The proportion of PCR positive animals was assumed to be a measure of the final size of the outbreak and used to estimate *R*_0_ for each outbreak (Table 3; see Additional file 10 for posterior densities for *R*_0_ for each outbreak). Posterior predictive checking showed that this model provided an acceptable fit to the data, with all observations lying in the 95% prediction intervals for each outbreak (see Additional file 11 for model checking). Across the outbreaks the median estimate for the basic reproduction number was higher for cattle (median: 0.90; range: 0.71–1.07) than for sheep (median: 0.71; range: 0.61–1.06) (Table 3). However, there was substantial overlap of the posterior distributions, indicating the estimates did not differ significantly between cattle and sheep. For all outbreaks, the 95% credible intervals for *R*_0_ included the threshold at *R*_0_ = 1. When the results for cattle and sheep were combined, the estimates for *R*_0_ were lower than those for the individual species (Table 3).

In addition to *R*_0_, the initial proportion of the household that was susceptible (*s*_0_) was also estimated for each outbreak (see Additional file 12 for posterior densities for *s*_0_ for each outbreak). Across the outbreaks the median initial proportion susceptible was the same for cattle (median: 0.82; range: 0.77–0.88) and sheep (median: 0.88; range: 0.76–0.93).

### Sampling at households with reported outbreaks

Additional environmental sampling was also carried out at three households with reported outbreaks in neighbouring LGAs, two in Wase LGA and one in Kanke LGA, both of which are in Plateau State (Figure 1B). Environmental swabs taken at all three households were positive for FMDV RNA (Table 4). All cattle from the three households had FMD-like lesions in their oral cavities (one bull from household P1, five cattle from household P2, and three cattle from household P7). Tissue samples were collected from five and two cattle from households P2 and P7 respectively, all of which tested positive by rRT-PCR and virus isolation. In addition, serum samples collected from sheep at all three households were positive by NSP ELISA or rRT-PCR (Table 4).

**Table 4:** **Sampling carried out at households with reported outbreaks**

Outbreak	Month sampled	Environmental Samples		Positive locations	Animal samples
		collected	positive		
Wase P1	September	4	4 (100%)	fence (× 2), feeding trough, rope	3/3 sheep NSP ELISA positive^a^
Wase P2	September	4	3 (75%)	fence, rope (× 2)	2/3 sheep NSP ELISA positive^a^
Kanke P7	December	5	2 (40%)	pegs, ropes	5/6 sheep rRT-PCR positive; 3/6 sheep NSP ELISA positive

^a^rRT- PCR results are not available for these animals.

Assuming the 5 (out of 6) samples from sheep positive by rRT-PCR (Table 4) are a measure of the final size for the outbreak in Kanke LGA, the basic reproduction number was estimated to be 2.05 (95% credible interval: 0.51–4.40) (Table 3).

### Sequencing

Eight samples were selected for sequencing using the probe enrichment technique; samples were selected to cover a range of C_T_ values, locations and sample types (animal and environmental) (see Additional file 1 for sample details). Sequences for the VP1 region of the FMDV genome were obtained for five of these samples which had C_T_ values that ranged between 20.9 and 26.6.

**Figure 6:**
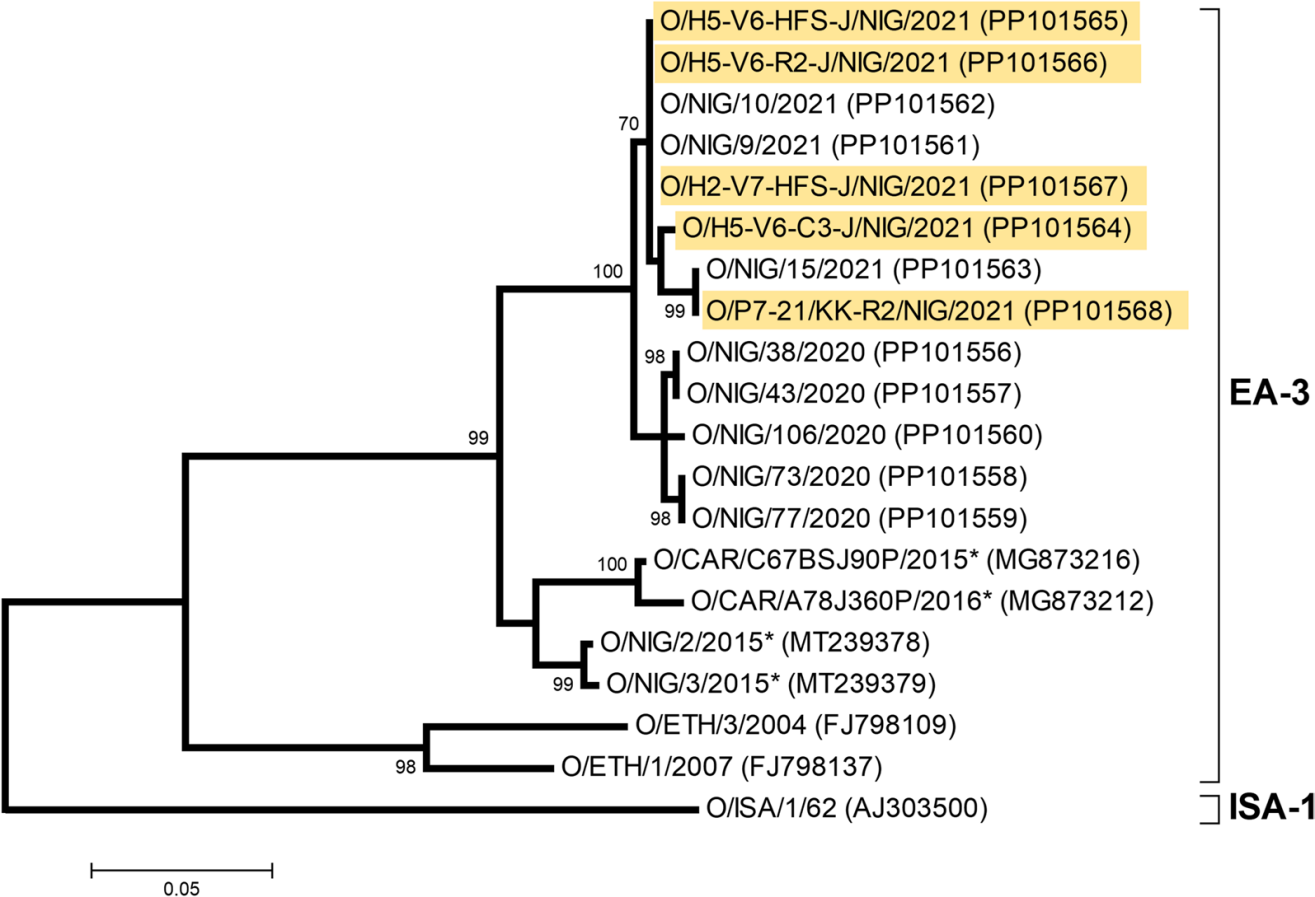
**Phylogenetic tree showing the relationship amongst foot-and-mouth disease virus VP1 sequences.** Sequences were generated from five samples collected during the present longitudinal study in northern Nigeria in 2021 (highlighted in yellow) and other sequences in the FAO World Reference Laboratory for FMD (WRLFMD) database. An asterisk (*) indicates virus designations not assigned by the WRLFMD.

All five samples for which VP1 sequence data were generated were serotype O, EA-3 topotype. There was a total of 633 positions in the final dataset. VP1 sequences were closely related to other samples collected in Nigeria and submitted to World Reference Laboratory for FMD (WRLFMD) over the same time period as the study (Figure 6). Samples H5-V6-R2-J/NIG/2021, H2-V7-HFS-J/NIG/2021, H5-V6-C3-J/NIG/2021 and H5-V6-HFS-J/NIG/2021 were all 99.7–100% identical to NIG/9/2021 (accession no. PP101561), whereas sample P7-21/KK-R2/NIG/2021 was 100% identical to NIG/15/2021 (accession no. PP101563) (Figure 6).

The VP1 sequences for the three environmental samples from the longitudinal study (two taken from household H5 in Jos South in September and one taken from household H2 in Jos South in October) were identical. The VP1 sequence for the serum sample taken from a bovine at household H5 in Jos South in September differed from the environmental samples taken at the same location. Finally, the VP1 sequence for the environmental sample collected at the outbreak in Kanke in December (Table 4) was more distantly related to those collected during the longitudinal study.

## Discussion

We conducted a longitudinal study to investigate the role that small ruminants and contaminated environments have on the endemicity of FMD, and the implications for disease transmission, surveillance and control.

We found that seropositivity varied amongst households and animal species, with a higher proportion of NSP ELISA positive samples in cattle, followed by sheep, and lowest in goats (Figure 4), which is aligned with findings in previous studies [5, 6, 9, 43, 44]. However, the seroprevalence did not change significantly over the study period (Figure 2). When the age-seroprevalence data were used to estimate the force of infection, we found that it was significantly lower in goats compared to sheep and cattle. By contrast, the force of infection was similar in sheep and cattle, suggesting sheep might play an important role in FMD transmission. The model used to estimate the force of infection assumes that FMDV is at equilibrium, which is reasonable given that the virus is endemic in the region. Although the strains and serotypes circulating have changed over time, one or more strains have been present in the study region [7, 8, 14]. Furthermore, because detection of antibodies against FMDV NSP reflects historic infection with any strain or serotype, it also means the force of infection is essentially related to an animal’s first exposure to FMDV, which may not be recent.

The force of infection inferred from age-seroprevalence data can be used to estimate the basic reproduction number (*R*_0_) by multiplying it by the mean lifespan of an animal [37]. This yield estimates for *R*_0_ for each household of between 1.00 and 2.31 for cattle and 0.39 and 0.89 for sheep in each household (Table 3), with the differences in *R*_0_ between species mostly a result of differences in longevity (mean of five and two years for cattle and sheep, respectively). The estimates of *R*_0_ for cattle are similar to those reported based on age-seroprevalence data from other endemic areas, for example, Ethiopia [45], but are much lower than is often reported for outbreaks in disease-free settings [46, 47].

In addition to using age-seroprevalence data we also estimated *R*_0_ for individual outbreaks based on rRT-PCR data, which measures only recent infection with any strain or serotype (for the target of the PCR used in this study). To do this we assumed the proportion of PCR positive cattle and/or sheep gave an estimate for the final size of the outbreak. For the four outbreaks in the earlier part of the study, this is reasonable as there was at least a three-month gap before other PCR positive animals were detected. For the six outbreaks that occurred later in the study, this may not be the case as PCR positive animals were detected when the last samples were taken in October. If the outbreaks had not finished, we would underestimate the final size and, hence, *R*_0_. Bearing this caveat in mind, the estimates for the basic reproduction number were mostly below one for both cattle and sheep, though the 95% credible intervals included the threshold at *R*_0_ = 1 (Table 3). An exception to this was the outbreak in Kanke for which *R*_0_ in sheep was estimated to be 2.05 (95% CI 0.51–4.40) (Table 3). The estimates for *R*_0_ are lower those obtained using outbreak data from Ethiopia [48], but are similar to those obtained using outbreak data from Thailand [49].

Our results have found little difference in the transmission of FMDV by sheep compared with by cattle, at least in our study setting. Only one study has quantified transmission from infected sheep to cattle under controlled conditions [50], which found the basic reproduction number of transmission from sheep to cattle (1.4) was similar to that for transmission from sheep to sheep (1.1), but was lower than that for transmission cattle to cattle. Further studies are needed to quantify transmission from small ruminants to other susceptible species to understand differences at host level.

The sheep and goats sampled in households were kept in the same premises and potentially had a similar level of exposure to FMDV, although anecdotal observations suggest that sheep and goats are kept separately, with sheep maintained in closer contact with cattle. A limitation of this study is that we did not collect detailed information regarding management practices. Further studies, with more detailed information about the management practices used in different geographic areas and production systems, are needed to better understand the extent to which this plays a role on the differences observed between seroconversion in sheep and goats and force of infection.

We also evaluated different sampling and testing methods in order to identify those which are reliable and convenient for surveillance in endemic settings. All oral swabs positive for FMDV RNA were collected during a month in which FMD outbreaks were reported (either in nearby locations or in one of the households that was part of the study). In contrast, no positive oral swabs were obtained during the months in which FMD outbreaks were not reported. These data suggest that FMDV positive oral swabs, as tested by rRT-PCR, are a good indicator of ongoing infection in a geographic region. Detecting FMDV RNA in serum was also evaluated as an alternative method. However, FMDV RNA in blood was found a few days after clinical signs were present and therefore it may be that animals recently infected were missed. However, FMDV RNA was detected in blood samples after the clinical window, demonstrating animals can still be infected after this time and clinical evaluation alone is a poor indicator of the presence of FMDV as viraemia is transient.

In addition, this study showed that environmental swabs are a good herd level indicator of FMDV circulating in the area. FMDV RNA was detected in environmental samples in all households that reported an outbreak. In addition, premises where FMDV RNA was detected in environmental samples as part of the longitudinal study had at least one animal in which FMDV RNA was detected in serum samples and/or oral swabs. This is aligned with previous studies that showed that environmental sampling can detect FMDV in a herd more quickly than clinical inspection if sufficient samples are collected often enough [20]. To increase the likelihood of detection of FMDV in the environment, it is important to sample at areas likely to have come into contact with secretions or excretions from infected animals. Our results suggest that good targets are boots, ropes, pegs, transport vehicles or hard floor surfaces.

Fragments of FMDV RNA may survive in the environment for prolonged periods of time [51], although persistence in the environment is likely to exhibit some seasonality based on changes in relative humidity and temperature [19]. As survival and persistence times for FMDV RNA in this environment are unknown, it is difficult to attribute positive samples to a particular outbreak in an endemic setting. However, our results suggest that the number of positive environmental swabs does match well with the number of positive oral swabs from animals. This suggests that an increase in the number of positive samples would be indicative of virus circulating in a region.

Our study showed that VP1 sequences could be obtained from environmental samples (including hard floor and ropes) with low C_T_ values (between 20.9 and 25.3). However, the RNA derived from environmental samples is likely to be fragmented and difficult to sequence by conventional methods. The incorporation of a probe enrichment step in a next generation sequencing library workflow enabled the recovery of viral genomic data from poor quality samples, as described previously [24]. When comparing the sequences obtained from environmental samples with sequences from other samples collected during outbreaks in nearby areas over the same time period as the study, we found that sequences obtained from environmental samples were closely related to those submitted from clinical samples. These findings confirm environmental sampling can be used as a surveillance method in endemic settings, especially when combined with sequencing.

This study sheds new light on the role of small ruminants on the epidemiology and endemicity of FMD. In addition, this study demonstrates the utility of oral and environmental swabs as suitable sampling methods for the early detection of FMDV infection at the animal and herd level, respectively. Furthermore, when combined with sequencing they allow for outbreak tracing.

## Supplementary Information

**Additional file 1.** **Samples selected for sequencing using a probe enrichment technique.**

**Additional file 2.** **Estimates for rRT-PCR sensitivity and specificity used to generate prior distributions.**

**Additional file 3.** **Shape parameters (α, β) for the beta distributions used as priors for sensitivity and specificity of rRT-PCR in oral swabs and serum samples.**

**Additional file 4.** **Deviance information criterion for binomial generalised linear mixed models for the probability of a positive sample.**

**Additional file 5.** **Serum rRT-PCR, oral swab rRT-PCR and NSP ELISA results for individual animals sampled at each household in Bassa LGA.** Each row in the three plots for each household corresponds to the same animal and is coloured yellow if the test result was negative, red if it was positive and grey if a sample of that type was not collected.

**Additional file 6.** **Serum rRT-PCR, oral swab rRT-PCR and NSP ELISA results for individual animals sampled at each household in Jos South LGA.** Each row in the three plots for each household corresponds to the same animal and is coloured yellow if the test result was negative, red if it was positive and grey if a sample of that type was not collected.

**Additional file 7.** **Deviance information criterion for models estimating the force of infection from age-dependent seroprevalence data.**

**Additional file 8.** **Concordance between results for oral swabs and serum samples taken from the same animals tested by rRT-PCR.**

**Additional file 9.** **Posterior predictive checking for the force of infection estimated from age-seroprevalence data.** Each plot shows the observed number of seropositive cattle or sheep (triangles) and the median (black line) and 95% range (shading) for the posterior predictive distribution.

**Additional file 10.** **Posterior densities for the basic reproduction number (R0) estimated for ten outbreaks in households in Bassa and Jos South LGAs.** Each plot shows the posterior density for the analysis based on cattle only (green), sheep only (cyan) or both cattle and sheep (magenta). The threshold at *R*_0_ = 1 is shown by a black dotted line.

**Additional file 11.** **Posterior predictive checking for the final size model used to estimate the basic reproduction number for outbreaks in households.** Each plot shows the observed number of cattle and/or sheep positive by rRT-PCR (bars) and the median (black diamonds) and 95% range (error bars) for the posterior predictive distribution.

**Additional file 12.** **Posterior densities for the initial proportion susceptible (s0) estimated for ten outbreaks in households in Bassa and Jos South LGAs.** Each plot shows the posterior density for the analysis based on cattle only (green), sheep only (cyan) or both cattle and sheep (magenta).

**Additional file 13.** **Full rRT-PCR and NSP ELISA results for all animal and environmental samples collected during the current study.**

## Data Availability

The data from the current study are available in Additional file 13.
