# Supplementary material for: Longitudinal study of foot-and-mouth disease virus in Northern Nigeria: implications for the roles of small ruminants and environmental contamination in endemic settings
Source: Vet Res. 2025 Apr 3;56:76. doi: 10.1186/s13567-025-01502-2 (PMC11969707; doi:10.1186/s13567-025-01502-2)
Supplement: Supplementary file 1 — Additional file 1. Samples selected for sequencing using a probe enrichment technique. [file 13567_2025_1502_MOESM1_ESM.docx]

**Additional file 1** **Samples selected for sequencing using a probe enrichment technique.**

| LGA | site† | month | sample type | C_T_ value | sequence |
| --- | --- | --- | --- | --- | --- |
| Bassa | H3 | October | sheep, oral swab | 32.1 | no |
| Jos South | H2 | October | environmental, hard floor surface | 23.5 | yes |
|  | H5 | September | environmental, hard floor surface | 20.9 | yes |
|  | H5 | September | environmental, ropes | 24.9 | yes |
|  | H5 | September | cattle, serum | 26.6 | yes |
| Kanke | P7 | December | environmental, ropes | 25.3 | yes |
| Wase | P1 | September | environmental, fence | 31.7 | no |
|  | P2 | September | environmental, ropes | 32.9 | no |

† H1-H5: households 1-5; LM - livestock market; T - transhumance location; P - reported outbreak
