# Supplementary material for: Longitudinal study of foot-and-mouth disease virus in Northern Nigeria: implications for the roles of small ruminants and environmental contamination in endemic settings
Source: Vet Res. 2025 Apr 3;56:76. doi: 10.1186/s13567-025-01502-2 (PMC11969707; doi:10.1186/s13567-025-01502-2)
Supplement: Supplementary file 2 — Additional file 2. Estimates for rRT-PCR sensitivity and specificity used to generate prior distributions. [file 13567_2025_1502_MOESM2_ESM.docx]

**Additional file 2** **Estimates for rRT-PCR sensitivity and specificity used to generate prior distributions.**

| Sample | Sensitivity | Specificity | Reference |
| --- | --- | --- | --- |
| Oral Pre-clinical  Clinical  Recovery | 0.89  0.92  0.82 | - | Nelson et al. [50] |
| Serum Pre-clinical  Clinical  Recovery | 0.75  0.82  0.64 | - | Nelson et al. [50] |
| Serum | 0.76 | 1.00 | Howson et al. [51] |
| Not specified | - | 1.00 | Callahan et al. [52] |
| Serum and oral swabs | - | 0.98 | assumed |
