# Supplementary material for: Longitudinal study of foot-and-mouth disease virus in Northern Nigeria: implications for the roles of small ruminants and environmental contamination in endemic settings
Source: Vet Res. 2025 Apr 3;56:76. doi: 10.1186/s13567-025-01502-2 (PMC11969707; doi:10.1186/s13567-025-01502-2)
Supplement: Supplementary file 3 — Additional file 3. Shape parameters (α, β) for the beta distributions used as priors for sensitivity and specificity of rRT-PCR in oral swabs and serum samples. [file 13567_2025_1502_MOESM3_ESM.docx]

**Additional file 3** **Shape parameters (α, β) for the beta distributions used as priors for sensitivity and specificity of rRT-PCR in oral swabs and serum samples.**

|  | Beta distribution (α, β) | |
| --- | --- | --- |
|  | Sensitivity | Specificity |
| rRT-PCR Oral swabs | 56.142, 7.902 | 65.102, 1.647 |
| rRT-PCR Serum | 26.189, 9.365 | 65.102, 1.647 |
