# Supplementary material for: Longitudinal study of foot-and-mouth disease virus in Northern Nigeria: implications for the roles of small ruminants and environmental contamination in endemic settings
Source: Vet Res. 2025 Apr 3;56:76. doi: 10.1186/s13567-025-01502-2 (PMC11969707; doi:10.1186/s13567-025-01502-2)
Supplement: Supplementary file 4 — Additional file 4. Deviance information criterion for binomial generalised linear mixed models for the probability of a positive sample. [file 13567_2025_1502_MOESM4_ESM.docx]

**Additional file 4.** **Deviance information criterion for binomial generalised linear mixed models for the probability of a positive sample.**

| model | deviance information criterion (DIC) |
| --- | --- |
| *rRT-PCR results, Bassa* |  |
| month | 68.9 |
| sample type* | 74.7 |
| month + sample type* | 71.1 |
| month + sample type* + interaction | 73.7 |
| month + sample type† + interaction | 67.7 |
| month + sample type‡ + interaction | 73.1 |
| *rRT-PCR results, Jos South* |  |
| month | 228.9 |
| sample type* | 299.8 |
| month + sample type* | 217.9 |
| month + sample type* + interaction | 211.7 |
| month + sample type† + interaction | 203.2 |
| month + sample type‡ + interaction | 211.7 |
| *ELISA results, Bassa and Jos South* |  |
| species | 729.7 |
| LGA⁋ | 845.6 |
| month + species | 730.7 |
| LGA + species | 729.8 |
| LGA + species + interaction | 720.7 |
| month + (LGA + species + interaction) | 721.2 |

* sample type coded as: environmental swab/oral swab/serum sample

† sample type coded as: environmental/cattle/sheep/goat

‡ sample type coded as: environmental swab/oral swab from cattle, sheep or goat/serum sample from cattle, sheep or goat (i.e. seven levels)

⁋ LGA: local government area (i.e. Bassa or Jos South)
