# Supplementary figures and images for: Longitudinal study of foot-and-mouth disease virus in Northern Nigeria: implications for the roles of small ruminants and environmental contamination in endemic settings
Source: Vet Res. 2025 Apr 3;56:76. doi: 10.1186/s13567-025-01502-2 (PMC11969707; doi:10.1186/s13567-025-01502-2)

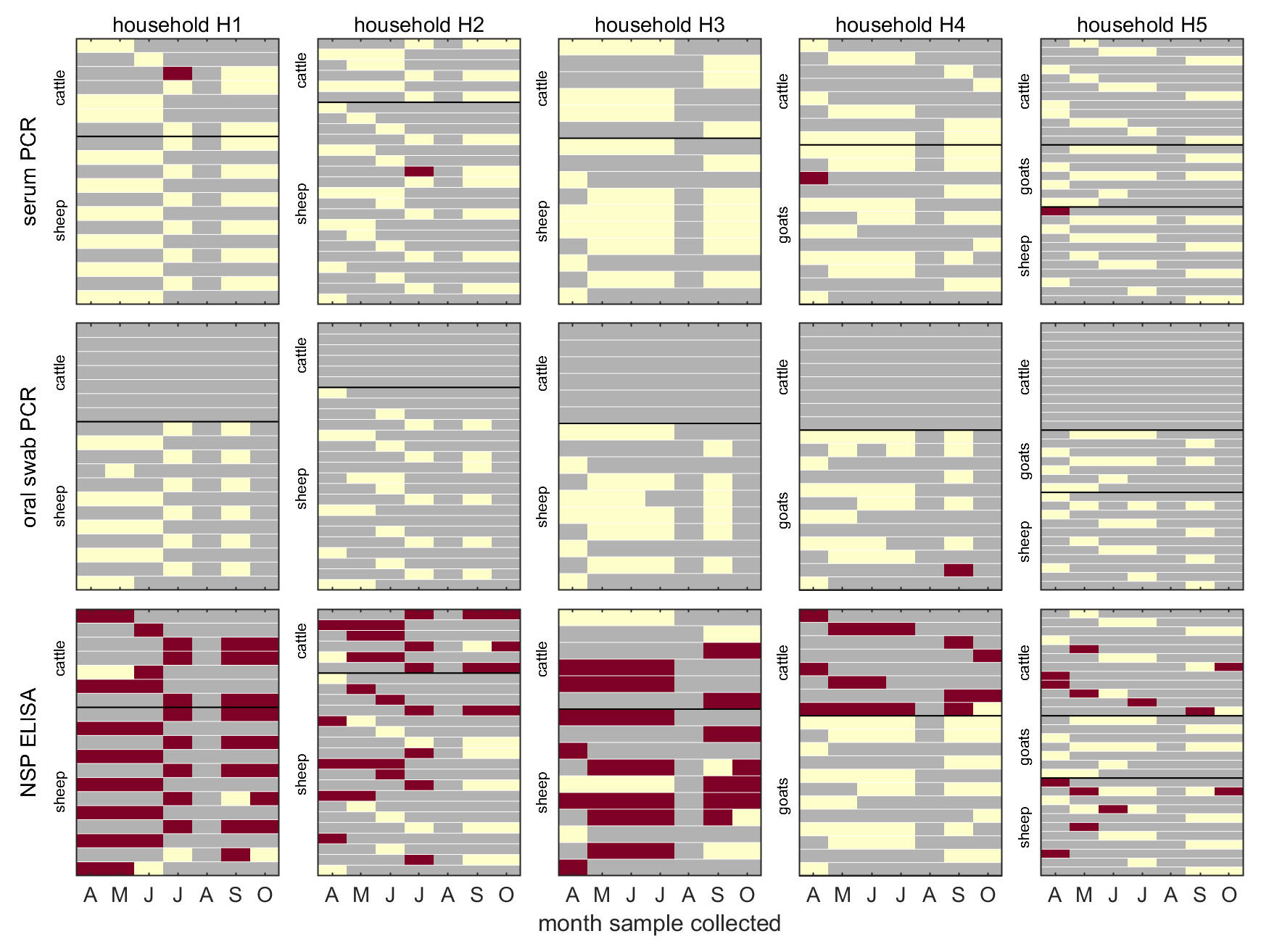

Supplement: Supplementary file 5 — Additional file 5. Serum rRT-PCR, oral swab rRT-PCR and NSP ELISA results for individual animals sampled at each household in Bassa LGA. Each row in the three plots for each household corresponds to the same animal and is coloured yellow if the test result was negative, red if it was positive and grey if a sample of that type was not collected. [file 13567_2025_1502_MOESM5_ESM.tif]

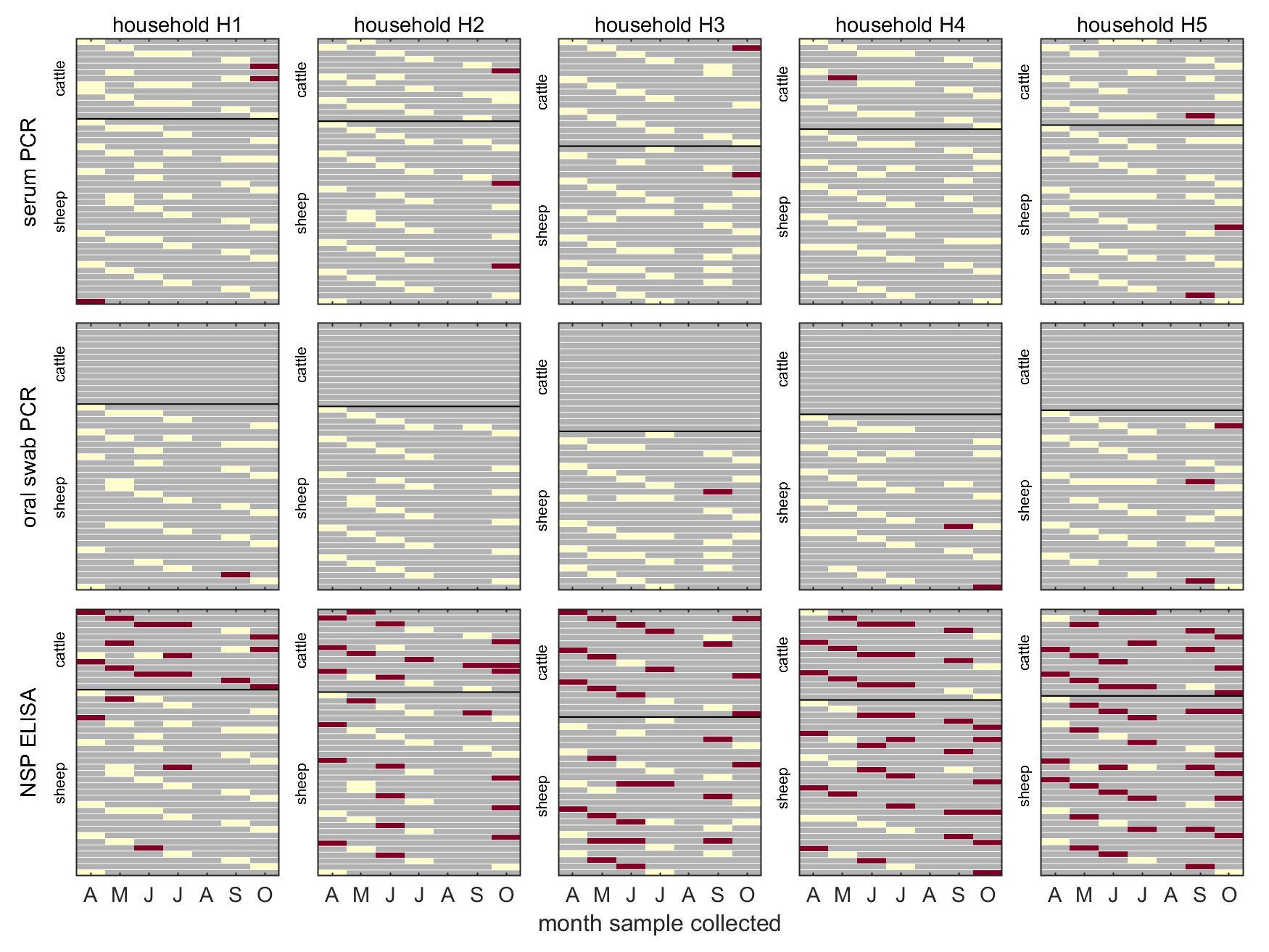

Supplement: Supplementary file 6 — Additional file 6. Serum rRT-PCR, oral swab rRT-PCR and NSP ELISA results for individual animals sampled at each household in Jos South LGA. Each row in the three plots for each household corresponds to the same animal and is coloured yellow if the test result was negative, red if it was positive and grey if a sample of that type was not collected. [file 13567_2025_1502_MOESM6_ESM.tif]

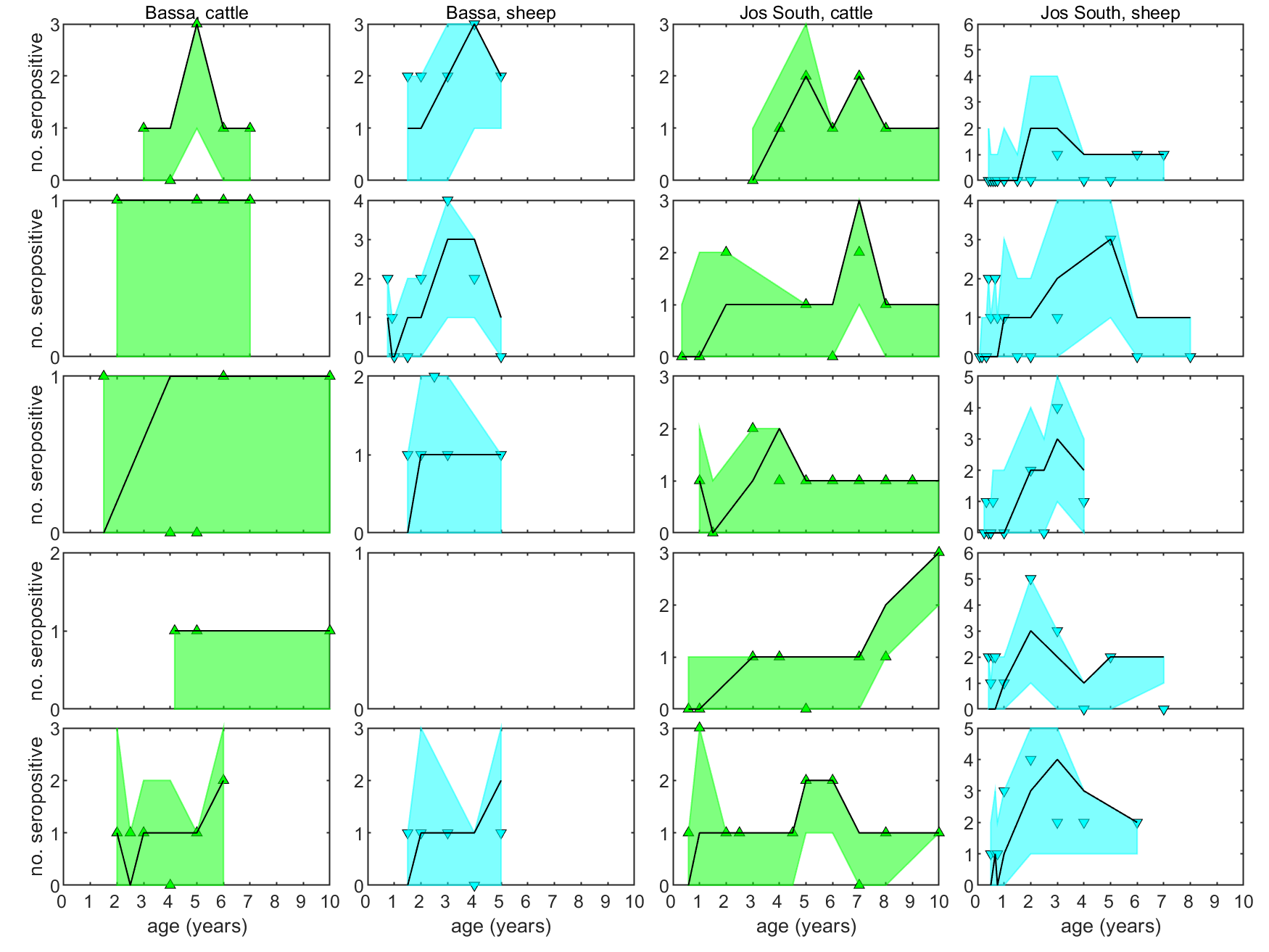

Supplement: Supplementary file 9 — Additional file 9. Posterior predictive checking for the force of infection estimated from age-seroprevalence data. Each plot shows the observed number of seropositive cattle or sheep (triangles) and the median (black line) and 95% range (shading) for the posterior predictive distribution. [file 13567_2025_1502_MOESM9_ESM.tif]

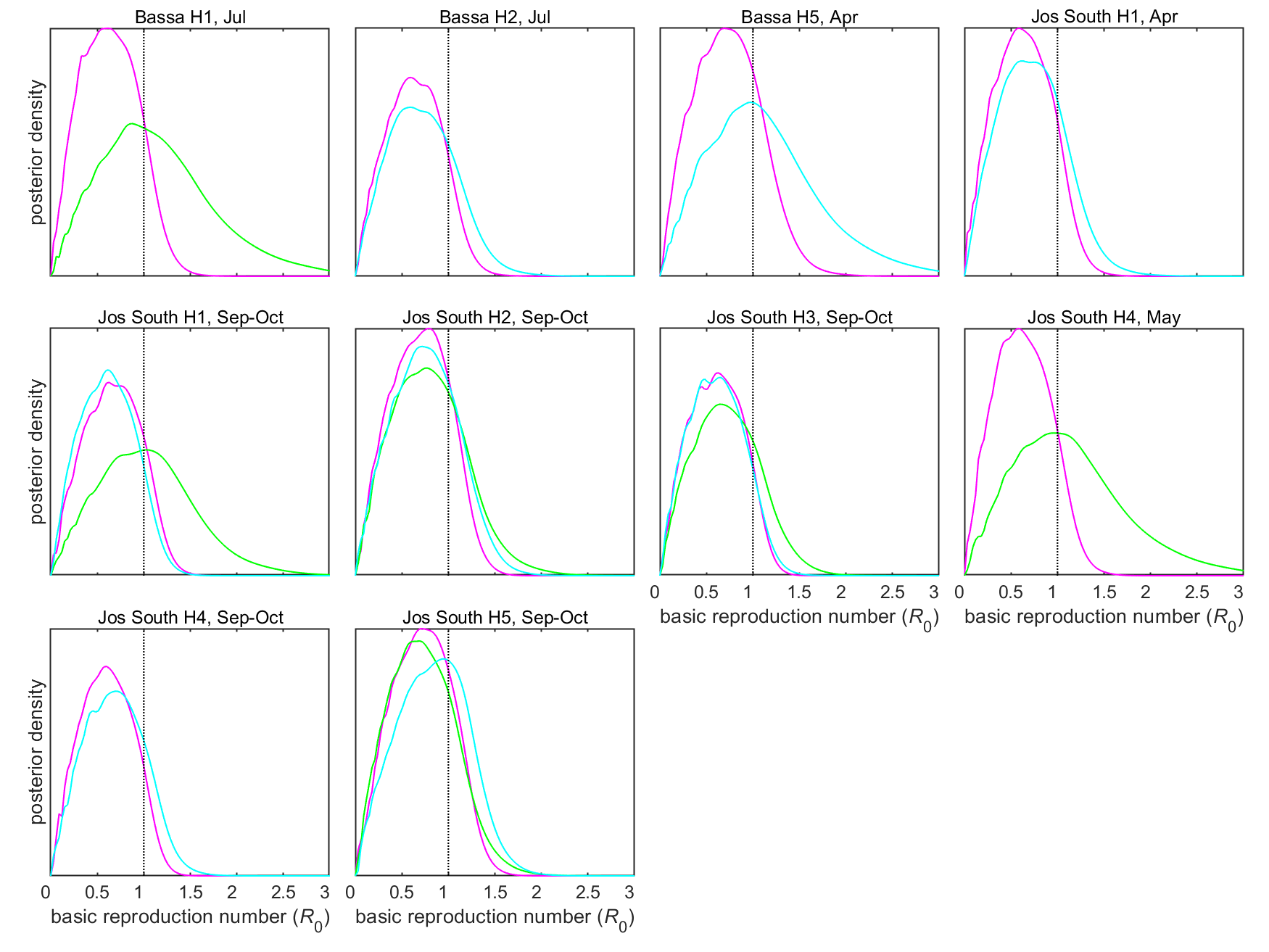

Supplement: Supplementary file 10 — Additional file 10. Posterior densities for the basic reproduction number (R0) estimated for ten outbreaks in households in Bassa and Jos South LGAs. Each plot shows the posterior density for the analysis based on cattle only (green), sheep only (cyan) or both cattle and sheep (magenta). The threshold at R0 = 1 is shown by a black dotted line. [file 13567_2025_1502_MOESM10_ESM.tif]

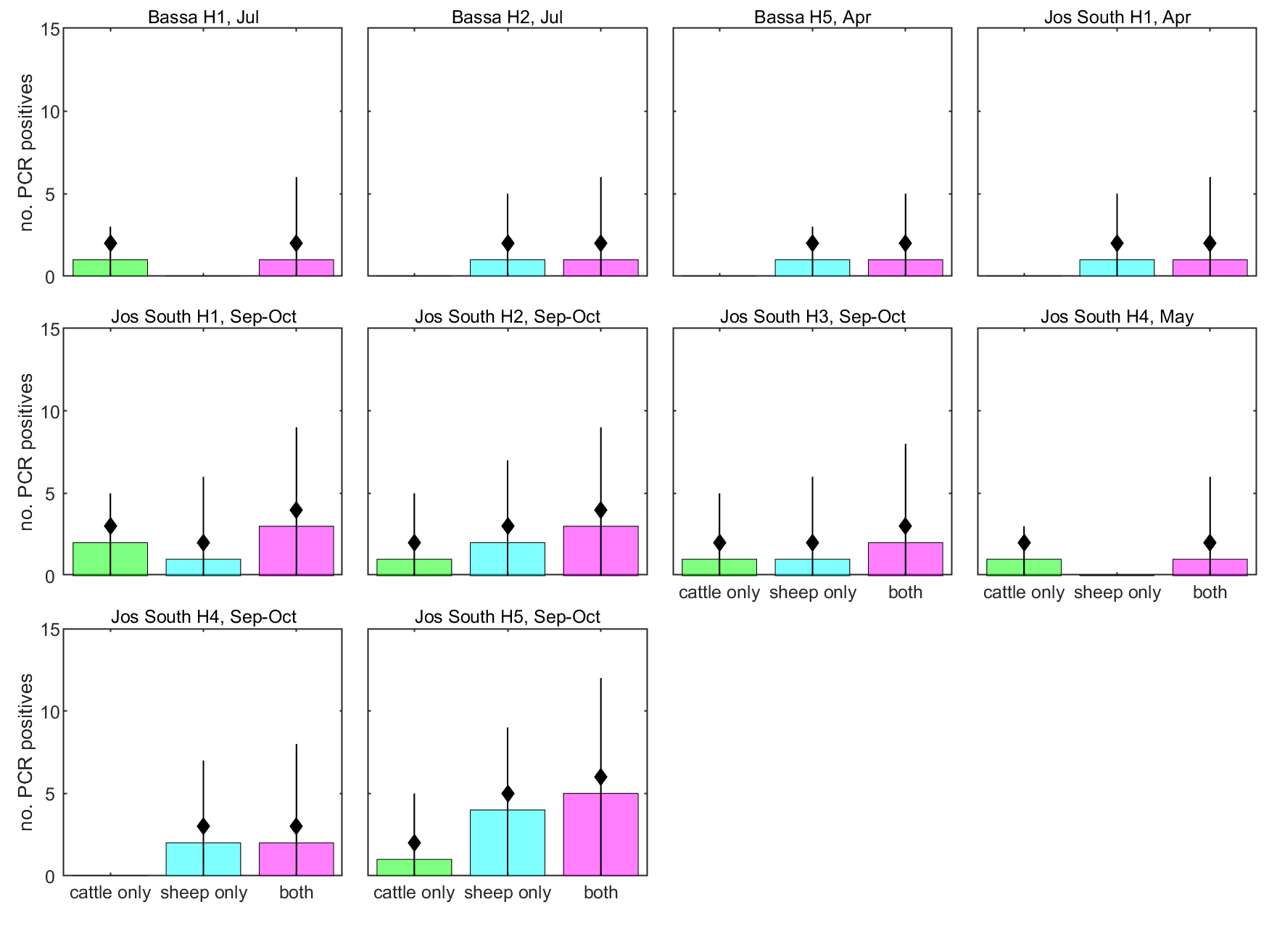

Supplement: Supplementary file 11 — Additional file 11. Posterior predictive checking for the final size model used to estimate the basic reproduction number for outbreaks in households. Each plot shows the observed number of cattle and/or sheep positive by rRT-PCR (bars) and the median (black diamonds) and 95% range (error bars) for the posterior predictive distribution. [file 13567_2025_1502_MOESM11_ESM.tif]

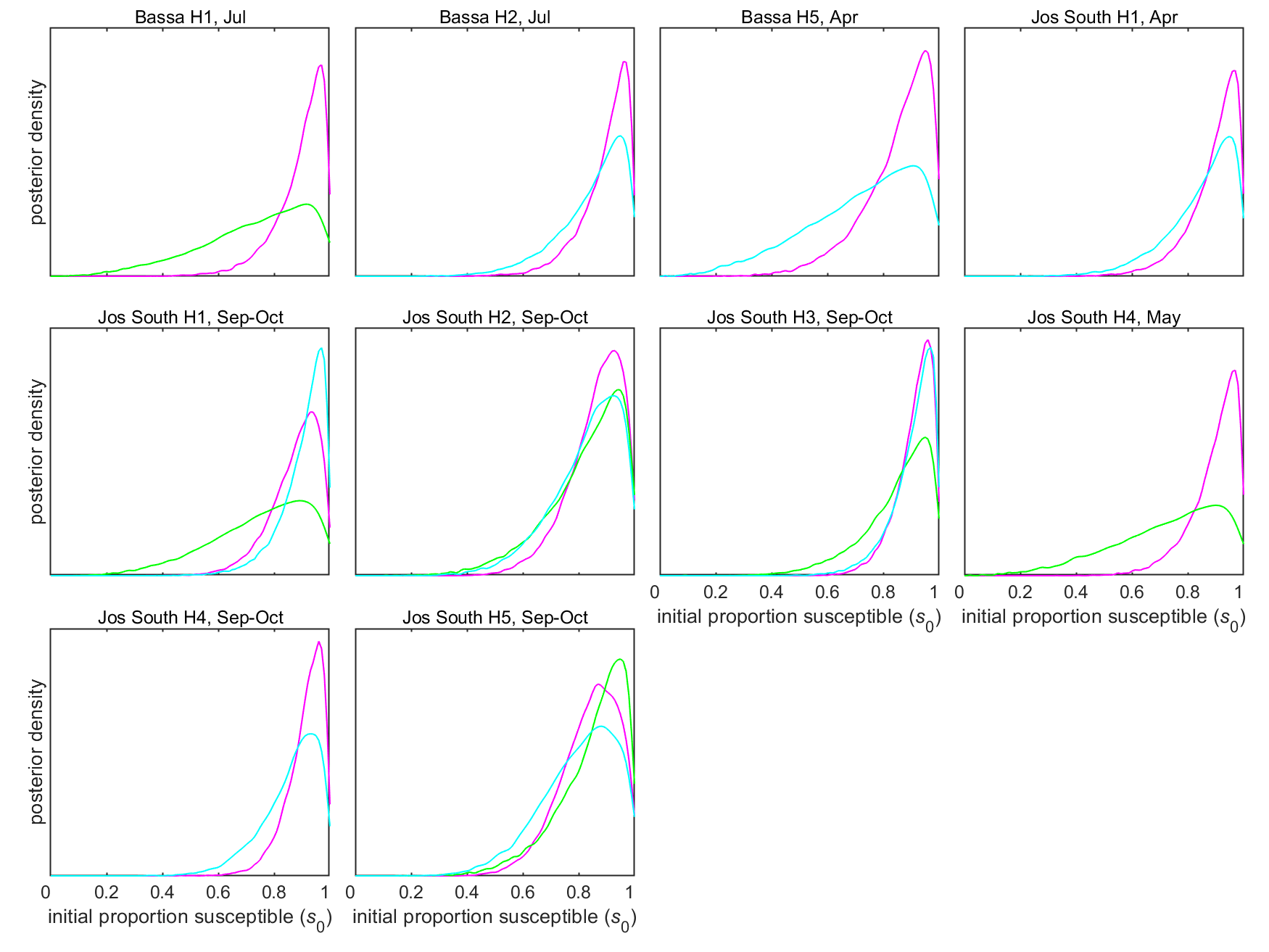

Supplement: Supplementary file 12 — Additional file 12. Posterior densities for the initial proportion susceptible (s0) estimated for ten outbreaks in households in Bassa and Jos South LGAs. Each plot shows the posterior density for the analysis based on cattle only (green), sheep only (cyan) or both cattle and sheep (magenta). [file 13567_2025_1502_MOESM12_ESM.tif]
