# Supplementary material for: Longitudinal study of foot-and-mouth disease virus in Northern Nigeria: implications for the roles of small ruminants and environmental contamination in endemic settings
Source: Vet Res. 2025 Apr 3;56:76. doi: 10.1186/s13567-025-01502-2 (PMC11969707; doi:10.1186/s13567-025-01502-2)
Supplement: Supplementary file 7 — Additional file 7. Deviance information criterion for models estimating the force of infection from age-dependent seroprevalence data. [file 13567_2025_1502_MOESM7_ESM.docx]

**Additional file 7** **Deviance information criterion for models estimating the force of infection from age-dependent seroprevalence data.**

| model | deviance information criterion (DIC) |
| --- | --- |
| baseline only | 403.4 |
| baseline + species | 384.9 |
| baseline + LGA*/household | 383.8 |
| baseline + species + LGA | 385.6 |
| baseline + species + LGA/household | 370.7 |

* LGA: local government area
