# Supplementary material for: Longitudinal study of foot-and-mouth disease virus in Northern Nigeria: implications for the roles of small ruminants and environmental contamination in endemic settings
Source: Vet Res. 2025 Apr 3;56:76. doi: 10.1186/s13567-025-01502-2 (PMC11969707; doi:10.1186/s13567-025-01502-2)
Supplement: Supplementary file 8 — Additional file 8. Concordance between results for oral swabs and serum samples taken from the same animals tested by rRT-PCR. [file 13567_2025_1502_MOESM8_ESM.docx]

**Additional file 8** **Concordance between results for oral swabs and serum samples taken from the same animals tested by rRT-PCR.**

| Serum | Oral swabs | | *P* value* | Kappa statistic |
| --- | --- | --- | --- | --- |
|  | Negative | Positive | 1 | 0.08 |
| Negative | 403 | 8 |  |  |
| Positive | 9 | 1 |  |  |

*McNemar test
